# Association of individual resilience with organizational resilience, perceived social support, and job performance among healthcare professionals in township health centers of China during the COVID-19 pandemic

**DOI:** 10.3389/fpsyg.2022.1061851

**Published:** 2022-11-29

**Authors:** An-Qi Wang, Chang-Hai Tang, Jia Song, Cheng-Xin Fan, Wan-Chen Wang, Zhong-Ming Chen, Wen-Qiang Yin

**Affiliations:** ^1^School of Public Health, Weifang Medical University, Weifang, Shandong, China; ^2^School of Public Affairs, Zhejiang University, Hangzhou, Zhejiang, China; ^3^School of Business, NingboTech University, Ningbo, Zhejiang, China; ^4^School of Management, Weifang Medical University, Weifang, Shandong, China

**Keywords:** individual resilience, organizational resilience, social support, job performance, primary healthcare professionals

## Abstract

**Background:**

Primary healthcare professionals were overworked and psychologically overwhelmed during the COVID-19 pandemic. Resilience is an important shield for individuals to cope with psychological stress and improve performance in crises. This study aims to explore the association of individual resilience with organizational resilience, perceived social support and job performance among healthcare professionals in township health centers of China during the COVID-19 pandemic.

**Methods:**

Data from 1,266 questionnaires were collected through a cross-sectional survey conducted in December 2021 in Shandong Province, China. Descriptive analysis of individual resilience, organizational resilience, perceived social support, and job performance was conducted. Pearson correlation analysis was used to examine the correlations among these variables, and structural equation modeling was performed to verify the relationships between these variables.

**Results:**

The score of individual resilience was 101.67 ± 14.29, ranging from 24 to 120. Organizational resilience (*β* = 0.409, *p* < 0.01) and perceived social support (*β* = 0.410, *p* < 0.01) had significant direct effects on individual resilience. Individual resilience (*β* = 0.709, *p* < 0.01) had a significant direct effect on job performance. Organizational resilience (*β* = 0.290, *p* < 0.01) and perceived social support (*β* = 0.291, *p* < 0.01) had significant indirect effects on job performance.

**Conclusion:**

During the COVID-19 pandemic, the individual resilience of healthcare professionals in township health centers was at a moderate level. Organizational resilience and perceived social support positively affected individual resilience, and individual resilience positively affected job performance. Furthermore, individual resilience mediated the effect of organizational resilience and perceived social support on job performance. It is recommended that multiple stakeholders work together to improve the individual resilience of primary healthcare professionals.

## Introduction

Individual resilience is described as the ability to absorb, adapt, bounce back, and maintain a stable equilibrium that unfolds in a context of adversity, failure, uncertainty, or dramatic changes (Bonanno, 2004; Luthans and Youssef, 2004; Karoly and Ruehlman, 2006). It represents positive adaptation and successful coping with stressful events and challenges (Sonn and Fisher, 1998). Individuals with resilience have the following recognized characteristics: a firm acceptance of reality, a life of deep beliefs and values, and a strong ability to improvise and adapt to significant changes (Luthans and Youssef, 2004). Individual resilience is also considered to be one of the key elements of positive psychological capital (Luthans et al., 2004). Individuals with high resilience adopt a positive attitude toward finding solutions to problems, whereas those with low resilience are susceptible to negative emotions and avoid problems, makingit difficult for them to bounce back from adversity (Ho and Chan, 2022).

Coronavirus disease 2019 (COVID-19), a severe acute infectious respiratory disease, was first reported in December 2019 and declared a pandemic by the World Health Organization (WHO) in early March 2020 (Duncan, 2020). The COVID-19 pandemic has posed serious challenges to healthcare systems globally, and primary care has become increasingly critical in responding to the COVID-19 pandemic because of the surge of patients and the inadequacy of treatment services for the population in secondary and tertiary hospitals (Haldane et al., 2020). Accordingly, the workload, operating conditions, and task structure of primary healthcare professionals have changed significantly (de Sutter et al., 2020). In China, nearly 4 million primary healthcare professionals have taken on many tasks beyond their usual duties during the COVID-19 pandemic (Zhou et al., 2020). Besides the increased workload and extended working hours, the risk of COVID-19 infection, lack of personal protective equipment, updated COVID-19-related treatment protocols, and separation from family have also posed a threat to the mental and psychological well-being of Chinese primary healthcare professionals, with more than half of them reporting symptoms of depression and anxiety during the COVID-19 pandemic (Ou et al., 2021; Shi et al., 2022). Persistent psychological problems can affect individuals’ overall health and lead to negative effects such as burnout and inefficiency at work, which can pose a threat to the integrity of the healthcare system and adequate care for patients (Fuchs et al., 2020; Wu et al., 2020). It was revealed that the psychological capital and job performance of healthcare professionals have become more vital than ever in these pandemic conditions (Pourteimour et al., 2021). Therefore, individual resilience, a protective factor in coping with stressful environments, alleviating negative emotions, preventing psychological disorders, and improving job performance cannot be overlooked (Luceño-Moreno et al., 2020).

Individual resilience is an important protective shield for healthcare professionals against psychological stress and mental breakdown during infectious disease outbreaks, and individuals who lack sufficient resilience are more vulnerable to negative psychological effects in a pandemic (De Brier et al., 2020). Moreover, individual resilience contributes to a strong psychological background for healthcare professionals to remain fully engaged in a pandemic, which has a positive impact on performance, and the adoption of resilience-enhancing strategies can improve the job performance of healthcare professionals (Handini et al., 2020; Jeong and Cho, 2020). Individual resilience in human resource management is stated as the ability to be competent for positions, emphasizing a stable psychological state and physiological function maintained by individuals in the context of adversity, which helps individuals to achieve good performance (Liang, 2013). Therefore, the matter of how to improve the individual resilience of primary healthcare professionals to cope with psychological stress and maintain satisfactory job performance during the COVID-19 pandemic is a widespread concern.

Existing studies have explored potential factors influencing individual resilience, such as demographic characteristics, personal attributes, personal skills and resources, and additional life stressors (Bonanno et al., 2007; Sousa et al., 2013; Huang et al., 2018). However, other potential factors (such as organizational resilience and social support) have rarely been explored. With little time for national healthcare systems to adequately prepare for the COVID-19 pandemic, organizational resilience is seen as being essential (Duncan, 2020). Focusing on individual resilience rather than organizational resilience is a way of missing the forest for the trees (Riess, 2021), as organizational resilience is an important foundation that can contribute to individual resilience, and shaping individual resilience is the mutual responsibility of the organization and the individual (Riess, 2021; Udod et al., 2021). In addition, studies interpreting the concept of individual resilience have included social support as one of the key attributes (Cooper et al., 2020). Studies have confirmed that perceived support from family, friends, and significant others during the COVID-19 pandemic, which makes individuals feel concerned and develop a sense of belonging, is regarded as an important protective factor to effectively handle and cope with various stressors in the working circumstances, and has a direct impact on individual resilience (Cooper et al., 2020; Labrague and De Los Santos, 2020; Shi et al., 2022). Thus, organizational resilience and perceived social support can be deemed to be important factors influencing the resilience of primary healthcare professionals during the COVID-19 pandemic.

Township health centers are an important part of the Chinese primary healthcare system, and also the hub of the Chinese three-tier healthcare system in rural areas (the rural three-tier healthcare system consists of county hospitals, township health centers and village health offices; Chen et al., 2021). By the end of 2020, China had 35.76 thousand township health centers, accounting for 3.69% of the total number of Chinese primary healthcare institutions (Website of the National Health Commission, 2021). Township health centers provide basic medical and public health services for rural residents, and are the “first line of defense” in guarding their health, as well as being an important cornerstone of the Chinese healthcare system (Sheng, 2018). Township health centers have taken on lots of the front-line work and public health responsibilities during the COVID-19 pandemic, including pre-screening and referral of fever cases, taking nucleic acid samples, performing health surveillance of high-risk groups, improving hospital infection control and personal protection, conducting vaccination, disinfecting the public environment, and educating the public on prevention of the pandemic (Zhou et al., 2022). In China, given the small number of healthcare professionals in township health centers and their limited experience in pandemic prevention and control, they have faced enormous psychological stress and performed unsatisfactorily during the COVID-19 pandemic (Meng et al., 2012; Ge, 2020; Yi et al., 2022). In this context, maintaining the resilience of rural primary healthcare professionals deserves great attention (Lei and Li, 2021).

Therefore, this study took healthcare professionals in township health centers as subjects, collected data on their resilience during the COVID-19 pandemic, and analyzed the relationships between individual resilience, organizational resilience, perceived social support, and job performance. We put forward the following hypotheses: (1) Organizational resilience and perceived social support are positively associated with the individual resilience of healthcare professionals in township health centers during the COVID-19 pandemic; (2) individual resilience is positively associated with job performance; (3) the relationship between organizational resilience and job performance, and the relationship between perceived social support and job performance are both mediated by individual resilience.

## Materials and methods

### Study design and sampling

The subjects of this study were healthcare professionals working in township health centers of China during the COVID-19 pandemic, and each of them read a statement describing the purpose of the survey and consented to engage in this survey. Before the formal survey, we conducted a pilot test in Weifang, Shandong Province, in September 2021 using simple random sampling. In total, 200 questionnaires were distributed and 186 valid questionnaires were collected, with a valid response rate of 93.00%. In the pilot test, the mean age of healthcare professionals in township health centers was 38.43 years old, 51.08% were women, and the Cronbach’s Alpha of the questionnaire was 0.977. In addition, the data collected from the pilot test were analyzed by Exploratory Factor Analysis (EFA), and four common factors were extracted (consistent with our prespecified four factors of individual resilience, organizational resilience, perceived social support, and job performance), the cumulative variance contribution was 67.555%, the factors loading ranged from 0.502 to 0.870, and the structural validity of the questionnaire was good. A formal cross-sectional survey with the stratified random sampling method to collect data on subjects was conducted in December 2021 in Shandong Province, which is an economically developed region in eastern China, and has 16 prefecture-level cities (a prefecture-level city is an administrative level below a province and above a county). First, three prefecture-level cities were selected in Shandong Province to represent economically developed cities, economically moderate cities, and economically underdeveloped cities. Then, three counties were randomly selected in each prefecture-level city, and four to six townships were selected in each county depending on the population size. Generally, there is one township health center in a township, and we sampled around 30 healthcare professionals for the anonymous self-administered questionnaire survey in each township health center. After removing invalid questionnaires with missing data that could not be supplemented and logical errors that could not be corrected, a total of 1,266 valid questionnaires were collected, with a valid response rate of 97.38%.

### Measurement instruments

#### Demographic characteristics

Demographic information collected in the questionnaire included gender, age, marriage, educational background, annual income, working tenure, average weekly working hours, etc.

#### Individual resilience

Referring to the resilience scale for rescuers in emergencies designed by Liang (2013), and following our thorough discussion and revision, the individual resilience scale in this study included 24 items. Each item required participants to rate it from 1 (strongly disagree) to 5 (strongly agree), and the scores of all items were summed to obtain a total individual resilience score (ranging from 24 to 120). The score on a five-point Likert item could be categorized into three levels: low (1.00–2.99), moderate (3.00–4.30), and high (4.31–5.00; Labrague and De Los Santos, 2020). We took this criterion and multiplied it by the number of items on variables, with scores for individual resilience of 24–71.99 indicating a low level, 72.00–103.20 indicating a moderate level, and 103.21–120 indicating a high level. These items of individual resilience were divided into four dimensions: rational coping including 6 items (describing the individual’s ability to remain sober and calm without being affected by emotions, and to analyze problems rationally and objectively when dealing with the COVID-19 pandemic and related events); hardiness including 5 items (describing the individual’s ability to keep faith and work hard to complete tasks in response to the COVID-19 pandemic and related events); self-efficacy including 7 items (description of individuals during the COVID-19 pandemic with successful experiences, being confident in their ability to handle emergencies effectively); and flexible adaption, with 6 items (describing the individual’s ability to accept adversity and be flexible and constructive in dealing with the COVID-19 pandemic and related events). The Cronbach’s Alpha for our sample in this scale was 0.967. The results of the Confirmatory Factor Analysis (CFA) found an acceptable fit for the model of this scale [Standardized Root Mean Square Residual (SRMR) = 0.038, Root Mean Square Error of Approximation (RMSEA) = 0.055, Adjusted Goodness of Fit Index (AGFI) = 0.912, Comparative Fit Index (CFI) = 0.970, and Tucker-Lewis Index (TLI) = 0.962; Deng et al., 2018; Heiss et al., 2020].

#### Organizational resilience

Organizational resilience was measured using the scale developed by Yan (2018), including two subscales: static resilience with 6 items (demonstrating the organizational risk prevention capabilities, flexibility, and collaboration capabilities) and dynamic resilience with 6 items (manifesting as capabilities of organizations to respond to, recover from, and gain experience and growth from the COVID-19 pandemic and related events). All items in this scale were rated on a five-point Likert scale, ranging from 1 (strongly disagree) to 5 (strongly agree), and the total score for organizational resilience was from 12 to 60. According to the above criterion (Labrague and De Los Santos, 2020), scores of organizational resilience ranging from 12 to 35.99, 36 to 51.60, and 51.61 to 60 indicated low, moderate, and high levels, respectively. The Cronbach’s Alpha for the present sample in this scale was 0.954. The results of the CFA found an acceptable fit for the model of this scale (SRMR = 0.019, RMSEA = 0.049, AGFI = 0.961, CFI = 0.992, and TLI = 0.987; Deng et al., 2018; Heiss et al., 2020).

#### Perceived social support

Perceived social support was measured with a twelve-item scale developed by Blumenthal et al. (1987). The scale was divided into three dimensions: perceived support from a significant other (4 items), perceived support from family (4 items), and perceived support from friends (4 items) during the COVID-19 pandemic. All items were rated on a five-point Likert scale, ranging from 1 (strongly disagree) to 5 (strongly agree), and the total score for perceived social support was from 12 to 60. According to the above criterion (Labrague and De Los Santos, 2020), scores of perceived social support ranging from 12 to 35.99, 36 to 51.60, and 51.61 to 60 indicated low, moderate, and high levels, respectively. The Cronbach’s Alpha for the present sample in this scale was 0.949. The results of the CFA found an acceptable fit for the model of this scale (SRMR = 0.021, RMSEA = 0.054, AGFI = 0.952, CFI = 0.989, and TLI = 0.980; Deng et al., 2018; Heiss et al., 2020).

#### Job performance

Motowidlo and Van Scotter (1994) divided job performance into task performance and contextual performance, and contextual performance was further divided into interpersonal facilitation and job dedication (Motowidlo and Van Scotter, 1994; Van Scotter and Motowidlo, 1996; Borman and Motowidlo, 1997). Referring to the scale developed by Motowidlo and Van Scotter (1994); Chen et al. (2021) designed a nine-item job performance scale tailored to the Chinese context (Chen, 2006), which was borrowed to measure the job performance of primary healthcare professionals in this study. This scale was divided into 3 dimensions with 3 items each: task performance (reflecting the direct contribution to organizational goals and core tasks, and measured mainly by the quantity and quality of the completed tasks during the COVID-19 pandemic), interpersonal facilitation (the performance in collaborating with, communicating with, or helping colleagues during the COVID-19 pandemic), and job dedication (taking the initiative to perform tasks outside the scope of their work and overcoming work difficulties during the COVID-19 pandemic, including self-discipline, proactive behavior, and hard work in support of organizational goals). Each item used a five-point Likert scale from 1 (strongly disagree) to 5 (strongly agree), with the total score for job performance ranging from 9 to 45. According to the above criterion (Labrague and De Los Santos, 2020), scores of job performance ranging from 9 to 26.99, 27 to 38.70, and 38.71 to 45 indicated low, moderate, and high levels, respectively. The Cronbach’s Alpha for the present sample in this scale was 0.911. The results of the CFA found an acceptable fit for the model of this scale (SRMR = 0.022, RMSEA = 0.056, AGFI = 0.962, CFI = 0.990, and TLI = 0.979; Deng et al., 2018; Heiss et al., 2020).

### Statistical analysis

First, descriptive analysis with frequencies and percentages was performed to describe the demographic characteristics of participants in this survey. Then, student’s *t-*test (*t*-test) and analysis of variance (ANOVA) were used to examine statistical differences across subgroups of demographic characteristics in scores on individual resilience, organizational resilience, perceived social support, and job performance. Pearson correlation analysis was conducted to estimate the correlations among these variables. IBM SPSS Statistics 21.0 was used for the above analyses. Finally, hypothesized relationships among individual resilience, organizational resilience, perceived social support, and job performance were validated with Structural Equation Modeling (SEM) on AMOS 21.0, using a bias-corrected bootstrap 95% confidence interval (CI) to further evaluate the significance of total, direct, and indirect effects (not including 0, significant). Additionally, fit indices of SRMR, RMSEA, AGFI, CFI, and TLI were proposed to assess model adequacy. We determined an acceptable fit using the conventional cut-off criteria of SRMR < 0.08, RMSEA < 0.08, AGFI > 0.90, CFI > 0.90, and TLI > 0.90 (Deng et al., 2018; Heiss et al., 2020). The significance level for all tests was determined as *p* < 0.05.

### Quality control

To control the data quality, the following steps were taken. In the study design stage, a formal questionnaire was formed by interpreting the relevant literature and using a pilot test. In the field research stage, the investigators were trained before the formal survey to ensure that they had a thorough understanding of the aim and content of the study. In the process of the formal survey, we assigned three to five specially trained investigators to each survey site. At least one investigator reviewed the quality of each questionnaire after it was completed. The investigators manually checked each item of the completed questionnaire to avoid omissions, mistakes, and logical errors. For example, if a healthcare professional’s age was 25 years old and his/her working tenure was written as 20 years, the investigator would re-interview the participant and ask him/her to correct it on the spot if there was a filling error, or if the same value was selected for all answers to the measured variables in the questionnaire, the investigator would ask the participant to reconfirm the accuracy of the answers. In the data collation and analysis stage, the questionnaires checked and passed were coded uniformly, and there was double-entry of data to control the quality.

## Results

### Demographic characteristics

Among the 1,266 healthcare professionals in township health centers who participated in this survey, most were females (70.62%) and married (83.33%). The mean age of these participants was 37.20 ± 9.20 years (ranging from 20 to 73 years old), and the majority of participants were in the age groups of 31–40 years (33.33%) and 41–50 years (34.52%). Most participants had a bachelor’s degree or higher (57.66%) and earned CNY 20,001–60,000 per year (64.61%). Nearly one-third (32.86%) of the participants have been working for more than 20 years. The majority of participants (58.29%) reported having worked 48 h or less per week. The specific information on demographic characteristics was shown in Table 1.

**Table 1:** Demographic characteristics of healthcare professionals in township health centers in this survey (*N* = 1,266).

Variables	*N* (%)	Variables	*N* (%)
Gender		Annual income (CNY)	
Male	372 (29.38)	≤20,000	93 (7.35)
Female	894 (70.62)	20,001–40,000	377 (29.78)
Age (years)		40,001–60,000	441 (34.83)
≤30	318 (25.12)	60,001–80,000	256 (20.22)
31–40	422 (33.33)	>80,000	99 (7.82)
41–50	437 (34.52)	Working tenure (years)	
>50	89 (7.03)	≤5	271 (21.41)
Marital status		6–10	239 (18.88)
Married	1,055 (83.33)	11–15	186 (14.69)
Unmarried/divorced/widowed	211 (16.67)	16–20	154 (12.16)
Educational background		>20	416 (32.86)
High school or below	150 (11.85)	Average weekly working hours (h)	
Associate’s degree	386 (30.49)	≤48	738 (58.29)
Bachelor’s degree or above	730 (57.66)	49–72	464 (36.65)
		>72	64 (5.06)

### Score differences across subgroups

The means of total scores for individual resilience, organizational resilience, perceived social support, and job performance among the 1,266 participants in this study were 101.67 ± 14.29, 51.29 ± 7.88, 49.10 ± 8.37, and 37.97 ± 5.22, respectively. The means of total scores for each dimension of the variables were detailed in Table 2.

**Table 2:** Means of total scores for individual resilience, organizational resilience, perceived social support, and job performance among the 1,266 participants in this survey (*N* = 1,266).

Variables	Mean	SD
Individual resilience		
Rational coping	23.61	4.51
Hardiness	22.14	3.14
Self-efficacy	30.56	4.27
Flexible adaption	25.38	4.08
Organizational resilience		
Static resilience	26.01	4.06
Dynamic resilience	25.28	4.23
Perceived social support		
Significant other support	16.07	3.08
Family support	16.74	2.94
Friends support	16.28	3.03
Job performance		
Task performance	12.29	1.98
Interpersonal facilitation	12.89	1.86
Job dedication	12.78	1.99

SD, Standard deviation.

Participants in different age groups, marital statuses and weekly working hours reported different scores for individual resilience and perceived social support. Participants who were older, married, and had shorter weekly working hours had higher scores for individual resilience and perceived social support. Meanwhile, those with moderate annual incomes had a higher organizational resilience score. Participants who were older, married, held bachelor’s degrees or above, had high annual incomes, and have been working over 20 years had a higher score for job performance. The different scores across subgroups of demographic characteristics were shown in Table 3.

**Table 3:** Score differences in individual resilience, organizational resilience, perceived social support, and job performance across different demographic characteristics groups in this survey (*N* = 1,266).

Variables	Individual resilience		Organizational resilience		Perceived social support		Job performance	
	Total scores	*t/F*	Total scores	*t/F*	Total scores	*t/F*	Total scores	*t/F*
Gender								
Male	102.55 ± 13.74	1.413	51.10 ± 7.94	−0.555	49.39 ± 8.25	0.792	38.15 ± 5.39	0.801
Female	101.31 ± 14.51		51.37 ± 7.85		48.98 ± 8.43		37.89 ± 5.15	
Age (years)								
≤30	99.92 ± 14.95	3.040^*^	51.11 ± 8.26	0.295	47.91 ± 9.20	3.060^*^	37.17 ± 5.24	4.381^**^
31–40	101.53 ± 13.55		51.50 ± 7.75		49.55 ± 7.91		37.92 ± 4.90	
41–50	102.61 ± 14.30		51.13 ± 7.69		49.34 ± 8.04		38.43 ± 5.19	
>50	104.02 ± 14.80		51.72 ± 8.08		50.02 ± 8.70		38.80 ± 6.31	
Marital status								
Married	102.05 ± 14.11	2.078^*^	51.21 ± 7.87	−0.812	49.45 ± 8.07	2.960^**^	38.19 ± 5.22	3.402^**^
Unmarried/divorced/widowed	99.81 ± 15.05		51.69 ± 7.88		47.36 ± 9.58		36.86 ± 5.06	
Educational background								
High school or below	102.17 ± 15.88	0.104	52.11 ± 7.17	1.789	50.15 ± 8.37	1.432	38.03 ± 6.07	4.216^*^
Associate’s degree	101.56 ± 14.81		51.60 ± 7.90		49.09 ± 8.75		37.34 ± 5.51	
Bachelor’s degree or above	101.63 ± 13.68		50.95 ± 7.99		48.88 ± 8.16		38.29 ± 4.83	
Annual income (RMB)								
≤20,000	97.55 ± 15.92	2.211	49.55 ± 9.45	3.017^*^	47.47 ± 8.65	1.018	36.55 ± 4.88	2.965^*^
20,001–40,000	102.17 ± 13.95		52.16 ± 7.50		49.34 ± 8.10		37.70 ± 5.29	
40,001–60,000	101.80 ± 14.57		51.26 ± 7.76		49.15 ± 8.72		38.22 ± 5.30	
60,001–80,000	102.33 ± 13.64		51.20 ± 7.90		49.31 ± 8.07		38.52 ± 4.83	
>80,000	101.42 ± 13.97		49.97 ± 7.77		48.90 ± 8.33		37.81 ± 5.59	
Working tenure (years)								
≤5	100.70 ± 14.21	2.100	51.70 ± 7.83	0.359	48.82 ± 8.68	0.662	37.08 ± 5.19	4.687^**^
6–10	100.67 ± 15.74		51.36 ± 8.16		48.58 ± 8.98		38.06 ± 5.59	
11–15	101.63 ± 13.72		51.20 ± 7.49		48.96 ± 7.90		37.70 ± 5.04	
16–20	100.69 ± 13.64		50.79 ± 8.21		49.29 ± 8.27		37.60 ± 4.67	
>20	103.27 ± 13.88		51.21 ± 7.81		49.57 ± 8.06		38.75 ± 5.18	
Average weekly working hours (h)								
≤48	102.29 ± 13.91	4.157^*^	51.53 ± 7.85	1.794	49.59 ± 8.09	4.424^*^	38.14 ± 5.06	1.161
49–72	101.34 ± 14.90		51.14 ± 7.90		48.64 ± 8.84		37.77 ± 5.48	
>72	97.06 ± 13.42		49.66 ± 7.91		46.78 ± 7.64		37.38 ± 5.00	

* *p* < 0.05.

** *p* < 0.01.

### Bivariate correlations

Individual resilience, organizational resilience, perceived social support, and job performance were correlated bilaterally. Individual resilience was positively related to organizational resilience (*r* = 0.619, *p* < 0.001), perceived social support (*r* = 0.634, *p* < 0.001), and job performance (*r* = 0.633, *p* < 0.001). Organizational resilience was positively related to perceived social support (*r* = 0.588, *p* < 0.001), and job performance (*r* = 0.438, *p* < 0.001). Perceived social support was positively related to job performance (*r* = 0.451, *p* < 0.001). More detailed correlation coefficients among the variables were presented in Table 4.

**Table 4:** Correlation coefficients of individual resilience, organizational resilience, perceived social support, and job performance in this survey (*N* = 1,266).

Variables	1	2	3	4	5	6	7	8	9	10	11	12
Individual resilience												
1. Rational coping	1											
2. Hardiness	0.646^***^	1										
3. Self-efficacy	0.658^***^	0.827^***^	1									
4. Flexible adaption	0.731^***^	0.713^***^	0.810^***^	1								
Organizational resilience												
5. Static resilience	0.503^***^	0.571^***^	0.555^***^	0.485^***^	1							
6. Dynamic resilience	0.528^***^	0.547^***^	0.533^***^	0.494^***^	0.804^***^	1						
Perceived social support												
7. Significant other support	0.535^***^	0.506^***^	0.521^***^	0.496^***^	0.531^***^	0.559^***^	1					
8. Family support	0.510^***^	0.560^***^	0.550^***^	0.514^***^	0.497^***^	0.515^***^	0.771^***^	1				
9. Friends support	0.547^***^	0.515^***^	0.526^***^	0.504^***^	0.481^***^	0.514^***^	0.792^***^	0.786^***^	1			
Job performance												
10. Task performance	0.443^***^	0.453^***^	0.529^***^	0.501^***^	0.346^***^	0.360^***^	0.352^***^	0.371^***^	0.362^***^	1		
11. Interpersonal facilitation	0.466^***^	0.546^***^	0.568^***^	0.532^***^	0.406^***^	0.365^***^	0.389^***^	0.434^***^	0.398^***^	0.668^***^	1	
12. Job dedication	0.447^***^	0.519^***^	0.560^***^	0.529^***^	0.402^***^	0.359^***^	0.346^***^	0.365^***^	0.354^***^	0.687^***^	0.751^***^	1

*** *p* < 0.001.

### Synthesized relationships verification by SEM

**Figure 1:**
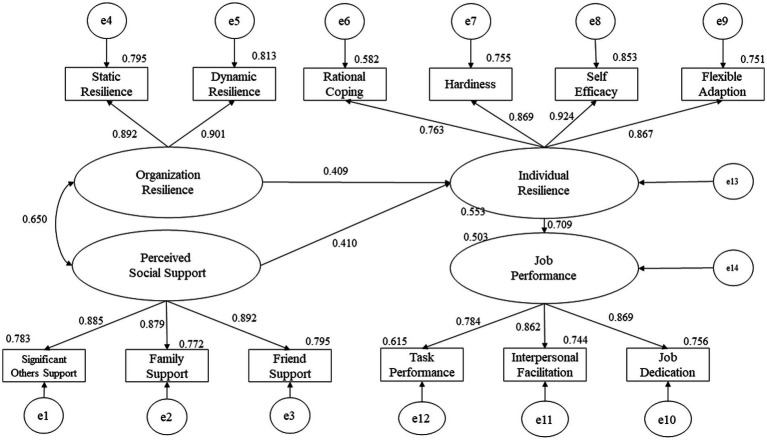
Model diagram of the synthesized relationships among individual resilience, organizational resilience, perceived social support, and job performance in this survey.

Figure 1 demonstrated the standardized path coefficients of the synthesized relationships among variables validated by SEM. The model shown in Figure 1 had a good fit (SRMR = 0.029, RMSEA = 0.074, AGFI = 0.922, CFI = 0.971, and TLI = 0.962). Table 5 with a bias-corrected bootstrap 95% CI presented the significance of the total, direct, and indirect effects. The path coefficients for all effects were significant. Organizational resilience [*β* = 0.409, 95% CI = (0.304, 0.495)] and perceived social support [*β* = 0.410, 95% CI = (0.323, 0.507)] had significant direct effects on individual resilience. Organizational resilience [*β* = 0.290, 95% CI = (0.222, 0.359)] and perceived social support [*β* = 0.291, 95% CI = (0.229, 0.371)] had significant indirect effects on job performance. Meanwhile, individual resilience [*β* = 0.709, 95% CI = (0.647, 0.759)] had significant direct effect on job performance.

**Table 5:** Total, direct and indirect effects among individual resilience, organizational resilience, perceived social support, and job performance in this survey (*N* = 1,266).

Paths	Total effects	95% CI	Direct effects	95% CI	Indirect effects	95% CI
Organizational resilience → Individual resilience	0.409^**^	[0.304, 0.495]	0.409^**^	[0.304, 0.495]	–	–
Organizational resilience → Individual resilience → Job performance	0.290^**^	[0.222, 0.359]	–	–	0.290^**^	[0.222, 0.359]
Perceived social support → Individual resilience	0.410^**^	[0.323, 0.507]	0.410^**^	[0.323, 0.507]	–	–
Perceived social support → Individual resilience → Job performance	0.291^**^	[0.229, 0.371]	–	–	0.291^**^	[0.229, 0.371]
Individual resilience → Job performance	0.709^**^	[0.647, 0.759]	0.709^**^	[0.647, 0.759]	–	–

** *p* < 0.01.

## Discussion

### Summary of main findings

This study was designed to explore the level of individual resilience of healthcare professionals in township health centers during the COVID-19 pandemic, and to analyze the relationships between individual resilience, organizational resilience, perceived social support, and job performance.

The score of the individual resilience for our collected sample was 101.67, showing a moderate level of resilience among healthcare professionals in township health centers during the COVID-19 pandemic, which was higher than the level of resilience reported in a study for primary care workers in Spain (Aragonès et al., 2022). Additionally, organizational resilience and perceived social support were positively associated with individual resilience, and individual resilience was positively associated with job performance, in line with the findings of previous studies (Hou et al., 2020; Riess, 2021; Shi et al., 2022). Moreover, the relationship between organizational resilience and job performance, and the relationship between perceived social support and job performance were both mediated by individual resilience.

### The effect of organizational resilience on individual resilience

Organizational resilience is related to the inherent and adaptive capabilities that enable organizations to detect threats, cope with disturbances, adapt and adjust to environmental changes, reduce organizational vulnerabilities to systemic risk environments, recover from adverse events, and even grow from them (Burnard and Bhamra, 2011; Jalil et al., 2021; Khalili et al., 2021). It can be understood from both static and dynamic perspectives, encompassing not only the capacity of organizations to proactively prevent risks, but also their capacity to cope with crises, bounce back from adversity, and survive in the long term (Zhang and Qi, 2021), which also has important implications for shaping individual resilience. The organizational resilience of the township health centers during the COVID-19 pandemic was at a moderate level, with a score of 51.29, and had a positive effect on individual resilience, which can be explained in the following ways.

First, resilient organizations are more likely to be taking steps before, during, and after crises to alleviate the psychological stress of healthcare professionals and enhance individual resilience (Young et al., 2021). Township health centers have been taking multiple steps during the COVID-19 pandemic to promote individual resilience by understanding the psychological status of primary healthcare professionals, monitoring individual stress responses, developing new support services and resources, rationalizing the working hours and rest times, and prioritizing individuals with special needs. Second, the organizational culture of resilience in township health centers can underpin the development of individual resilience among primary healthcare professionals. The features of leadership, effective communication, efficient policy implementation, empowerment, solidarity and cooperation, and altruism available in healthcare organizations during the COVID-19 pandemic have been noted to be associated with a culture of resilience at the organizational level, contributing to the resilience of individuals (Wu et al., 2020; Brown et al., 2021; Fleuren et al., 2021). Third, township health centers with resilience pay more attention to improving the work environment, such as through the provision of appropriate personal protective equipment and disinfection of hospital communal facilities, which alleviates anxiety about COVID-19 infection among primary healthcare professionals. The association between low anxiety levels and high resilience scores among healthcare professionals has been shown to be strong (Sakr et al., 2022).

### The effect of perceived social support on individual resilience

Perceived social support is a subjective perception and assessment of the degree of support received from one’s external surroundings, such as family, friends, and significant others (Zimet et al., 1990). It allows for solving problems or regulating emotions arising from stressful events by providing tangible assistance (such as medical assistance, financial support, and useful information) or emotional values (such as empathy and belonging; Mo et al., 2020; Hofman et al., 2021). The perceived social support of healthcare professionals in township health centers during the COVID-19 pandemic was at a moderate level, with a score of 49.10, and perceived social support positively affected individual resilience, further explaining previous studies that found social support to be an important resource for healthcare professionals improving their ability to cope with stress during the COVID-19 pandemic (Aughterson et al., 2021).

Some studies reported that healthcare professionals were not fully prepared for emergencies, including the COVID-19 pandemic (Labrague et al., 2018; Labrague and De Los, 2021). Inadequate preparation can make it difficult for healthcare professionals to take on the physical and psychological stresses of a pandemic in a short period, showing vulnerability. Instrumental and informational support from significant others (such as leaders and experienced colleagues) in terms of emergency response methods, risk communication skills, and critical information findings can bridge gaps in crises preparedness for healthcare professionals and promote rational coping with and flexible adaptation to the COVID-19 pandemic and related events, which would enhance individual resilience among healthcare professionals in township health centers. Furthermore, given the fast spreading nature of COVID-19, healthcare professionals in township health centers have been forced to isolate themselves from family, friends and society, which left them feeling anxious and depressed. Emotional support from family and friends can promote positive emotions, increase confidence, alleviate anxiety and depression, and enhance hardiness and self-efficacy with individual resilience for them (Ortiz-Calvo et al., 2022; Shi et al., 2022). Additionally, work-life balance is an essential attribute of resilience (Cooper et al., 2020). Healthcare professionals in township health centers have put lots of effort into their work during the COVID-19 pandemic, with limited time allocated for family life. Family members can support healthcare professionals in achieving a work-life balance by sharing more family responsibilities, enabling primary healthcare professionals to improve their resilience.

### The effect of individual resilience on job performance

Job performance of healthcare professionals, a key objective of organizational management, refers to their behavioral presentations and outcomes in healthcare work and is profoundly associated with individual resilience (Liu et al., 2019; Hoşgör and Yaman, 2022). The job performance of healthcare professionals in township health centers during the COVID-19 pandemic was at a moderate level, with a score of 37.97, and was affected by individual resilience.

Resilience protects individuals against various negative emotions and prevents poor psychological states such as stress, depression, and anxiety, thereby improving the professional skills and job engagement of primary healthcare professionals with a positive effect on job performance (Ojo et al., 2021; Yörük and Güler, 2021; Hoşgör and Yaman, 2022). Studies have also shown that resilient individuals are able to take control of themselves, thus avoiding transgressions in the workplace and contributing to increased productivity (Song, 2012). These viewpoints can serve to explain the positive relationship between the individual resilience and job performance of healthcare professionals in township health centers during the COVID-19 pandemic. Moreover, resilience is regarded as being related to a sense of coherence, which was defined as individuals having a dynamic feeling of confidence in being able to cope with stressful challenges (Streb et al., 2014). A strong sense of coherence enables individuals to mobilize the available resources for effective coping with stress and avoiding burnout (Levert et al., 2000), which plays a positive role in improving the job performance of healthcare professionals in township health centers. We also found that individual resilience mediated both the relationship between organizational resilience and job performance, and the relationship between perceived social support and job performance.

### Implications for practice

The individual resilience of healthcare professionals in township health centers requires long-term investment and sustained attention, rather than no longer valued as crises abate, and it should involve the joint efforts of individuals, organizations, health authorities, and society. Primary healthcare professionals have been required to acquire increased specialized knowledge and emergency management skills to adapt rapidly and cope rationally with the COVID-19 pandemic and related crisis events, and to learn from experiences in practice to improve their individual confidence and self-efficacy. Meanwhile, healthcare professionals are encouraged to relieve negative emotions through reflective journaling, thoughts sharing, and mindfulness training, to develop hardiness and promote individual resilience by accepting their limitations, finding meaningful objects in life, and learning to grow in life experiences (Bonanno, 2004).

Building organizational resilience and fostering the individual resilience of primary healthcare professionals should be a priority for healthcare organizations. Firstly, organizations should take the COVID-19 pandemic as an opportunity to improve the capacity to monitor and predict risks, develop emergency plans and conduct simulation exercises, and strengthen organizational teamwork, with the aim of crisis prevention and preparedness. Secondly, organizations are expected to bounce back rapidly from crises and achieve a new development stage. Thirdly, during a pandemic, organizations should develop the individual resilience of primary healthcare professionals at the organizational level by monitoring their health status, providing technical guidance, offering targeted support programs and creating an optimal working environment. Additionally, in the early stages of a pandemic, organizations need to redeploy staffing structures, form a flexible shift schedule, optimize the workflow, and guarantee adequate rest time for primary healthcare professionals (Johal et al., 2021), with a view to improving individual adaptation to the stressful environment, attending to their the mental state and enhancing resilience. What’s more, it is recommended that health authorities and society provide instrumental and emotional support for healthcare professionals during the pandemic to enhance resilience in terms of sharing information, stocking, providing adequate emergency supplies, and offering medical support and emotional value when necessary.

### Limitations and future research

Although this study provides an important reference for enhancing the individual resilience of primary healthcare professionals during the COVID-19 pandemic, some limitations were noted. Firstly, the data in this study were from one province in eastern China, rather than being representative of the national situation, so we will consider taking one province in central China and one province in western China in a future study, to obtain a more comprehensive understanding of the resilience of healthcare professionals in township health centers during the COVID-19 pandemic. Secondly, several limitations were imposed by the nature of the study design. The cross-sectional survey meant that causal relationships between variables could not be proven, and the adoption of self-reported methods for collecting data implied that social desirability effects due to observation bias were unavoidable. In future studies, we will incorporate a combined qualitative and quantitative approach for the study design and the collection of sample data from multiple subjects. Finally, we expect to include more individual factors (such as demographic information, personal characteristics, and resources available to the individual) in the analysis of factors influencing individual resilience.

## Conclusion

This study focused on exploring the individual resilience of healthcare professionals in township health centers during the COVID-19 pandemic, and found that individual resilience was at a moderate level. Moreover, organizational resilience and perceived social support were positively associated with individual resilience, and individual resilience was positively associated with job performance; furthermore, individual resilience mediated the relationship between organizational resilience and job performance, and the relationship between perceived social support and job performance. Synthesizing the findings of this study, it is recommended that enhancing resilience from various perspectives, including individual professional skills and psychological quality improvement, organizational resilience enhancement, material security and emotional support, which involves the joint efforts of primary healthcare professionals, healthcare organizations, health authorities and society.

## Data availability statement

The original contributions presented in the study are included in the article, further inquiries can be directed to the corresponding authors.

## Ethics statement

Ethical review and approval was not required for the study on human participants in accordance with the local legislation and institutional requirements. Written informed consent from the patients/participants or patients/participants legal guardian/next of kin was not required to participate in this study in accordance with the national legislation and the institutional requirements.

## Author contributions

A-QW, C-HT, JS, C-XF, and W-CW were responsible for the conception and design, drafting the manuscript, reviewing, and editing the paper. A-QW and C-HT were responsible for evaluating and analyzing data. Z-MC and W-QY critically reviewed and commented on the draft paper. All authors contributed to the article and approved the submitted version.

## Funding

This study was funded by the National Natural Science Foundation of China (grant numbers: 71804131 and 72274140), “Youth Innovation and Technology Program” Project of Colleges and Universities in Shandong Province (grant number: 2020RWG014), the Humanities and Social Science Foundation of the Ministry of Education of China (grant number: 22YJAZH137), and the Key Research and Development Program of Shandong Province (Soft Science Project) (grant number: 2022RKY07002).

## Conflict of interest

The authors declare that the research was conducted in the absence of any commercial or financial relationships that could be construed as a potential conflict of interest.

## Publisher’s note

All claims expressed in this article are solely those of the authors and do not necessarily represent those of their affiliated organizations, or those of the publisher, the editors and the reviewers. Any product that may be evaluated in this article, or claim that may be made by its manufacturer, is not guaranteed or endorsed by the publisher.
